# Functionality Review of Mobile Apps for the Tracking and Self-Management of Fatigue: Systematic Search in App Stores and Content Analysis

**DOI:** 10.2196/84755

**Published:** 2026-08-11

**Authors:** Amr Diouf Abdulla, Corina Sas, Gavin Doherty

**Affiliations:** 1 School of Computing and Communications, Lancaster University, Bailrigg, Lancaster, LA1 4YW, United Kingdom, 44 1524594541; 2 School of Computer Science and Statistics, Trinity College Dublin, Dublin, Ireland

**Keywords:** chronic fatigue syndrome, fatigue, mobile apps, tracking, self-management, interventions, pacing

## Abstract

**Background:**

Fatigue and chronic fatigue syndrome (CFS) have a considerable impact on quality of life, thus motivating people to develop skills for better management of their fatigue. While the number of commercial apps in this domain has increased, there has been limited exploration of their functionalities.

**Objective:**

This paper aims to address this research gap through a functionality review of 17 top-rated iOS and Android apps for fatigue, with the aim to articulate design implications for technologies focused on supporting the management of fatigue.

**Methods:**

We conducted a systematic search on the 2 most common app marketplaces, which resulted in the initial identification of 427 Apple apps and 1218 Google apps. From these, 17 apps were selected for review after applying a screening process to shortlist the top-rated apps. The functionalities of these apps were then coded through a week-long usage of each app for an expert evaluation leveraging authors’ human-computer interaction (HCI) expertise. We looked for functionalities such as tracking and visualization seen in previous research on functionality reviews, in addition to interventional functionalities, which were informed by research on fatigue.

**Results:**

Findings reveal the prevalence of functionalities for tracking fatigue (8/17, 47%), related symptoms (8/17, 47%), for visualizing tracked content (10/17, 59%), for assessing the user’s condition (2/17, 12%), and for providing interventions for the management of fatigue (12/17, 71%). Functionalities providing interventions for self-management of fatigue are surprisingly limited, with the most relevant ones including pacing (2/17, 12%) alongside energy estimation (2/17, 12%).

**Conclusions:**

The top-ranked apps for fatigue in the major marketplaces support 3 main functionalities under the scope of tracking fatigue along with related data, and visualizing such data, with limited provision of self-management interventions. Drawing from these findings, we articulate implications for the sensitive design of technologies to support the management of fatigue, including supporting hybrid tracking, combined visualizations to support sense-making of fatigue data with related factors, and supporting energy estimates and pacing interventions.

## Introduction

### Background

#### Overview

Fatigue and particularly chronic fatigue syndrome (CFS) are conditions with a strong impact on quality of life. Crook et al [1] define fatigue as “more profound than being overtired; it is unrelenting exhaustion and a constant state of weariness that reduces a person’s energy, motivation, and concentration.” Within human-computer interaction (HCI) research on physical health, fatigue has been less explored, although recent work, focused on lived experiences of long COVID, has started to emerge [2]. In parallel to academic research, there has been a growing number of mobile apps targeting fatigue. Mobile apps for physical or mental health, and in particular, those highly rated by their users, are likely to reflect valuable design principles which benefit from being articulated as implications for design. Similar efforts have been made for apps for depression [3], digital well-being [4,5], and anxiety [6], but less so for fatigue. To address this gap, we report a functionality review of 17 top-rated apps for fatigue from the Apple App Store and Google Play Store, together with an analysis of their descriptions on marketplaces. Findings indicate that the main functionalities of these apps are tracking fatigue and its symptoms, visualizing tracked content, assessing symptom impact on everyday life, and providing interventions. We conclude with 5 design implications for better support of people living with fatigue, which include supporting hybrid tracking to balance the validity of self-reported fatigue with low-burden automatic tracking of symptoms, contextualizing fatigue data with symptoms and associated factors through combined visualizations for sense-making, and supporting energy estimates and pacing interventions.

To support our exploration and understanding of the functionalities, we draw on the limited but emerging area of HCI work on fatigue, comprising the tracking of symptoms, the design of technologies supporting their management, and the emerging area of research that has engaged in the analysis of apps created for this purpose.

#### HCI Studies Around Technology Usage for Individuals With Chronic Conditions

A key area that we first needed to understand was the behaviors of individuals with CFS surrounding their use of technology to have a better idea of any ramifications that may arise from the condition, and consequently how they can be designed around. Paymal and Homewood [7] conducted a study to identify how CFS impacts technology usage in day-to-day life, in which they make use of their findings to present design recommendations. Their findings include the reduction of technology use on days where participants feel better, the increased usage of technology to help with tasks when symptoms were more noticeable, the avoidance of technology by some participants when their symptoms were more severe, and the usage of apps for tracking and self-management purposes. While the researchers identified how self-management technologies were used by this group, their reasons for turning to these technologies, and their criticisms regarding their usage experience, the researchers’ findings could be further supported by detailing the different functionalities offered.

Virtual reality (VR) is also an area that has been the focus of research; Best and Butler [8] investigated the use of VR as a support space for individuals with CFS. They discovered that CFS symptoms would prevent users from engaging with the technology for extended periods of time; however, this led to users improving their energy management to use their limited reserves more efficiently, in addition to optimizing their physical spaces and the technology they use to minimize fatigue triggers. This highlights the interplay between their energy levels and their self-tracking efforts, and is a key area of concern when designing for this group of users.

Davies et al [9] investigated the experiences of patients with chronic fatigue regarding their usage of technology for disease management, and a key area of interest was the participants’ usage of apps for self-management. In their study, they set forth design guidelines that are centered around the symptoms that patients want to manage the most, including features that assist users in identifying maximum and minimum energy thresholds, adding activity pacing functionality coupled with routine establishment, providing support for fatigue tracking by exploring automated logging methods to reduce effort expended, and finding ways to make self-management apps more engaging and motivating such as by gamification. Their findings can better inform our evaluation as the guidelines provide a frame of reference to examine the apps against.

#### Understanding Technologies for Individuals With CFS

Research has also delved into understanding how to support users with energy-limiting conditions (ELCs) through technology. For instance, Børsting and Culén [10] applied a sense-making process for complex domains in order to explore the design of technologies for supporting young individuals with CFS. The approach is a combination of a literature review, mapping techniques, and user research. The results of the user research, which was conducted with CFS experts, led to suggestions targeting self-management, namely, providing interventions discovered by other individuals with CFS, offering reminders for both resting and general purposes, providing functionalities to track and warn users of noise levels, providing social support technologies, and finally, providing the logging of symptoms coupled with tracking technologies suited for CFS.

Another strand of work has focused on technologies for supporting the management of chronic conditions. For instance, Sas et al [2] reported findings from workshops with individuals affected by long COVID, suggesting design implications for tracking and sense-making of fatigue and postexertional malaise (PEM), as well as self-management aimed at PEM prevention. Their findings stressed the importance of lightweight capture of data to prevent symptoms worsening among users, conjointly with reducing the burden of identifying both triggers and the PEM-approaching stage by making use of machine learning (ML) techniques or noninvasive items such as heart rate variability (HRV) tracking, and finally, supporting self-management of fatigue based on the stage of PEM that a user is at.

We also note the study of Homewood and colleagues [11] who co-designed through asynchronous communication with the aim of catering to the needs of individuals with ELCs. The researchers discovered that this method helped participants to only engage with the study when their energy levels were sufficient to partake; in addition, they were able to extract meaningful insights into improving the support for individuals with ELCs. These included designing technologies that improve communication with their social networks, implementing automatic tracking of their fatigue levels and associated symptoms, and using crip approaches in future studies dealing with ELCs.

Studies have also found that if participants’ fatigue symptoms reached more severe levels, they may avoid interaction with technology to prevent overwhelming them [7,9,12]. For this reason, designing around the potential fluctuations in their energy levels is paramount [2,9,10,13,14], and one such way this can be achieved is by including automated logging methods, especially in scenarios where users track numerous types of data [2,9]. In the same vein, Mack et al [14] proposed tenets to improve the accessibility of technology, and from these, one tenet called for designs that account for rises in fatigue levels post exertion and consider the consequences of a task on an individual’s energy reserves.

The findings detailed in this section can be used to better evaluate the current design space of commercial apps and provide more informed implications for future app designs.

#### Better Design of Online Resources for Individuals With CFS

Other areas of HCI research targeting chronic conditions have focused on the usage of online resources to support CFS, as in the study of Brigden et al [15]. This study, along with several others, found that social media could be of value to individuals with chronic conditions as a means of discussion and social support surrounding their condition [7,10,15-17]. These studies highlight the importance of observing for social media–based functionalities in the apps to be reviewed.

Another study is by Best and Butler [12], where they sought to identify the physiological effects experienced by individuals with CFS when socializing in a virtual world. Their findings also include the previously mentioned user behavior where they withdraw from technology as their fatigue levels worsen, so as to prevent further deterioration [7,9,12]. As such, the level at which users must interact with the apps could be a useful metric to evaluate the apps and think of further design implications.

#### HCI Research on Technologies for Long COVID

PEM is also known to be a characteristic of conditions such as long COVID [2]; therefore, the research in this field has value for the area of fatigue management. From the works in this field, we note the study of Homewood [13], who undertook an autoethnography where they used a Fitbit device for activity pacing purposes to manage their long COVID symptoms. This is also one of the studies that highlighted the importance of designing around the potential fluctuations in energy levels [2,9,10,13,14].

Another study of interest is that of Homewood et al [16], who conducted interviews with participants who had long COVID and made use of wearables for disease management, with findings highlighting the need for designs that are tailored to account for individual differences.

In addition, Pater and colleagues [17] carried out a 3-month-long cohort study on patients with long COVID, which involved the collection of both objective and subjective data. The authors emphasized that cognitive symptoms may make data gathering difficult, with participants finding it challenging to enter survey data regularly due to forgetfulness, which also made interviews burdensome [17]. They also posited that gaps in data tracked during their study were caused by participants forgetting to charge their wearable devices.

Finally, Mehdipour and Aharari [18] conducted a literature review along with case studies to analyze the marrying of the fields of AI, HCI, and Internet of Things (IoT)—which is defined by Kelly et al [19] as “a system of wireless, interrelated, and connected digital devices that can collect, send, and store data over a network without requiring human-to-human or human-to-computer interaction”—in regard to the analysis of data surrounding COVID. Their findings highlight that these fields aided the process of understanding large health datasets built from the data of numerous individuals.

These findings reveal the need for flexibility in tracking options to prevent overwhelming the user, and this exploration can be aided with HCI principles.

#### HCI Research on Technologies for Chronic Pain

We also draw from research on chronic pain, as it is a condition for which self-tracking is used for self-management. Adams and colleagues [20] investigated preferences regarding the self-assessment of pain for the purpose of self-managing chronic pain, through which they also discovered the previously mentioned importance of tailoring designs to suit individual differences [16,20].

Singh et al [21] carried out a study to explore the use of movement sensing and sonifying wearables by individuals with chronic pain in their undertaking of functional activity. The researchers found that the device challenged the user’s initial beliefs on their limits by improving their bodily awareness, and this led to users feeling more confident in trialing activity pacing methods. This highlights the importance of tracking and identifying limits for their self-management efforts.

#### Personal Informatics for the Support of Chronic Conditions

Personal informatics is an interdisciplinary research area within the broader HCI discipline, focusing on how people use personal technologies in life to collect, organize, and use information about their daily lives, in order to support personal goals in a range of domains such as productivity, learning, well-being, and health [22]. In the space of chronic conditions, this research area contributes design principles for digital health technologies such as mobile apps aimed at supporting fatigue. HCI work on chronic conditions has focused on tracking symptoms [23,24] or data related to physical activity [22,25,26], heart rate [27,28], sleep [22,29], and various other general health aspects [23,24,30].

Personal informatics also has value in supporting interventions; for instance, Felipe et al [29] found that it can greatly supplement the intervention of pacing, which is commonly undertaken by individuals with ELCs [2,9] and/or chronic pain [29,31]. In addition, individuals with chronic migraines also made use of personal informatics to identify if their condition is worsening in order to take preventative measures [32], which is known to be an important aspect of pacing [2].

Moreover, personal informatics can help users with chronic health conditions understand their condition better [24,29,32], offload the burden of monitoring their condition [29], improve communication and discussions with clinicians [24,32], help identify when their condition worsens, which is essential for taking preventative action in time [32], and finally, aid users in managing and monitoring their condition in the long term [32].

### Objectives

Despite the numerous works focused on chronic conditions, limited research has drawn design implications for supporting fatigue by exploring the breadth of functionalities offered by top-rated apps in this space. In our research, we will highlight how our findings compare with existing apps for supporting the management of chronic fatigue syndrome, in terms of items tracked and interventions. To this end, we put forth the following research objectives:

1. Conduct a systematic search on the top-rated commercial apps supporting fatigue
2. Explore and detail the range of functionalities offered by these apps
3. Identify whether the apps inform users of their theoretical or empirical underpinnings
4. Discern how the apps handle user data through the app’s privacy policies and cookie notices
5. Inform design guidelines drawing from the functionalities discovered

We aim to address that gap by conducting a systematic search and a functionality review on the top-rated apps from today’s marketplaces. To inform the methodology and paper structure, this paper draws on work done on app reviews in the areas of mindfulness [33], depression [34], goal setting [35], and digital wellbeing [4].

## Methods

### Overview

This section presents the process of selecting the apps and the description of functionality review, as well as analysis of app description and privacy terms.

### Apps Selection Process

We conducted a systematic app search, for which we used a PRISMA (Preferred Reporting Items for Systematic Reviews and Meta-Analyses) search strategy [36], similar to previous research on app reviews [4,33-35,37-39] including some in JMIR journals [4,34,38,39]. The search was conducted in the autumn of 2024 on the app’s store descriptions, using the keywords “chronic fatigue syndrome,” “chronic fatigue syndrome,” “fatigue,” “ME/CFS,” “CFS,” “myalgic encephalomyelitis,” and “pace or pacing.” This search was completed on the 2 most common marketplaces, which is also consistent with prior app reviews [4,33-35,37-39].

From the returned apps, we removed duplicates from both platforms, and for the remaining apps, we applied an inclusion criterion of having an average rating score higher than 3.0 and a number of ratings higher than 10. By screening the resulting apps, we further excluded apps that did not use any of the keywords or used them irrelevantly, as well as those not in English or requiring a subscription to use without even a free trial.

We chose to review apps that are top-rated (rating scores above 3.0) as they reflect positive user experiences of interacting with the apps, in addition to having a robust set of functionalities that the users appreciated and most likely found useful. From an HCI perspective, exploring these functionalities is beneficial as we can infer design principles and identify how we can improve the future design of such apps [4,33-35,37]. In addition, we chose to review apps that are free to access, similar to prior work [4,33,35,40], as they are more likely to provide better accessibility to the general population and fairness, as some users may not be able to afford the subscriptions [41], which is especially important in the health care context. In addition to being free, these apps might also have functionalities that are problematic, which our review aims to identify.

### Functionality Review

As noted by Hossain et al [42], functionality review is an increasingly common HCI method used to explore current technologies and their main features, particularly commercial mobile apps [4,33-35], which authors identified as being descriptive, that is, merely describing the functionalities, or critical, that is, involving deeper analysis and integration [42].

For this, the first author engaged with the apps twice a day for at least 1 week, using both an Android 14 version phone and an iPhone iOS 18.1.1 version phone. This ensured that each app was used for a total cumulative time ranging from 70 to 140 minutes. The second author reviewed 3 out of 17, for a duration of 3 days. This was to add independent codes, ensure the codes are clear and consistently used by independent raters, and validate the coding process. The proportion of apps assigned to the second coder is consistent with similar studies such as the review of goal apps [35] and the review on depression apps [34].

The coding process was hybrid [43], involving both deductively identified functionalities such as tracking [33-35], visualizing [33-35], and interventions [33,34] for self-management, common in previously reported HCI work on app reviews, while the inductively identified ones emerging from the reviewed apps included subfunctionalities regarding the content being tracked, such as fatigue and its symptoms. We also coded characteristics such as the theoretical and scientific underpinnings of the app’s functionalities plus their privacy policies and cookie notices. When coding the former, if we did not find the information pertaining to this while using the app, we sought it in the app’s help pages and websites.

With regard to interventions for fatigue management, Davies et al [9] highlighted the importance of “pacing” in order to help people remain within limits of their energy level, promoting routinization, and tending to their mental health. Pacing has also been found to be used in complement with the participant’s energy management efforts [9]. For this reason, the authors searched for functionalities specifically revolving around these concepts as part of their coding.

Interrater agreement—which was done only on the codes concerning tracking and interventional functionalities due to the large number of codes—using Cohen κ [44] showed almost perfect agreement for 149 out of 188 (79.3%) codes, fair agreement for 5 out of 188 (2.7%), and slight agreement on 34 out of 188 (18.1%) codes. The grouping and labeling of these codes into almost perfect, fair, and slight agreement were informed by the study of Landis and Koch [45]. From these, the authors revised the codes with fair or lower agreement.

The codes with slight agreement were mostly related to the tracked items captured along with their modalities. The main reason for the difference was due to a greater usage length being required to fully capture all the codes, especially with regard to the vast number of items tracked along with their recording frequencies. As such, information gleaned from the longer usage duration was used to revise the codes into agreement.

### Ethical Considerations

No human subjects participated in the research. For apps offering social media functionalities, we did not analyze any user-submitted content. Neither primary nor secondary human-related data were collected for this submission, which focuses exclusively on the description of the apps found on marketplaces and their functionalities.

## Results

### Overview

The app selection process resulted in a final set of 17 top-rated commercial apps from the Apple App Store and the Google App Store. Findings highlight that the identified apps not only include tracking functionalities but also support interventions for the management of fatigue, which involves managing energy levels and preventing crashes. The identified functionalities can be grouped into the main categories of tracking, visualizing, assessing, providing interventions, and ensuring privacy. Tracking functionalities comprise the collection of fatigue data and other related symptoms. Functionalities for visualization aim to represent the data in a manner that the user can understand and use toward the management of their condition. Assessment functionalities gauge the impact of the user’s symptoms through validated scales. Intervention functionalities aim to help users manage their condition, and this can involve assisting with energy management and crash prevention. Finally, in the section highlighting the app’s privacy measures, we will identify how apps handle users’ data, the usage of cookies along with their purpose, and the age groups targeted. The full codebook has been supplied in the form of a Microsoft Excel sheet for viewing all codes and most of the subcodes (Multimedia Appendix 1).

### App Selection Results

**Figure 1.**
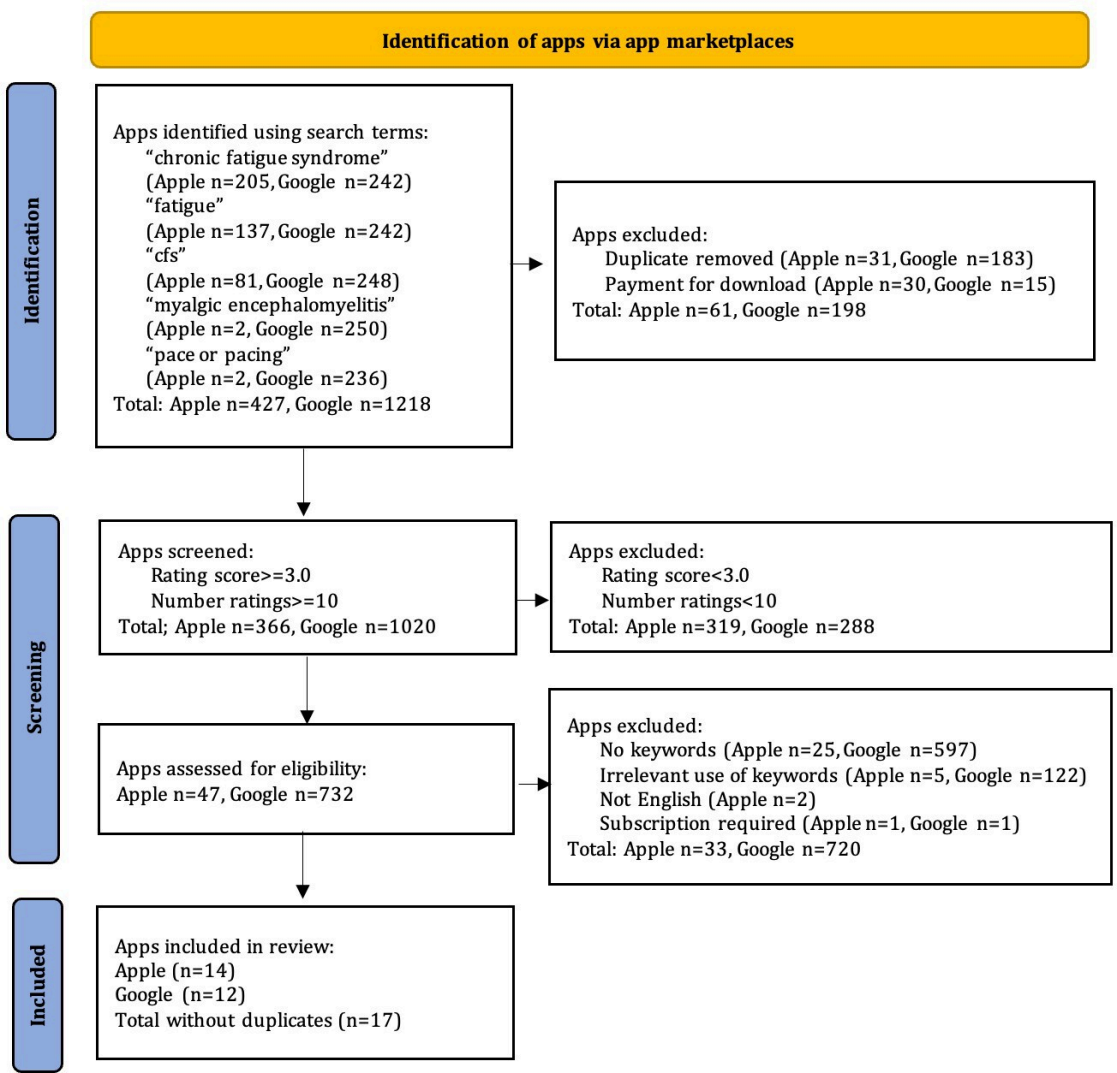
PRISMA (Preferred Reporting Items for Systematic Reviews and Meta-Analyses) diagram for app extraction, detailing inclusion-exclusion criteria alongside app numbers included or excluded at each stage.

The initial search returned 427 apps on the Apple App Store and 1218 on the Google Play Store (Multimedia Appendix 2), and from these, we removed 214 duplicates from both platforms. Applying our inclusion criteria resulted in 366 apps from the Apple App Store and 1020 from the Google Play Store. By screening these, we further excluded apps that were less relevant, required a subscription, or were not in English, leading to 14 Apple apps and 12 Google apps. After accounting for duplicates across the 2 platforms, our final set comprised 17 apps: 5 from the Apple App Store, 3 from the Google Play Store, and 9 from both. The PRISMA diagram in Figure 1 [36] shows the selection process.

### App Characteristics

A summarized view of the app characteristics has been presented in Multimedia Appendix 3.

While all selected apps had rating scores above 3, most apps from both stores had rating scores above 4.0 (13/14 for Apple apps and 10/12 for Google apps). Among the Apple apps, 4 apps had more than 100 reviews, and 2 apps had more than 1000 reviews. Most of the selected Google apps had more than 100 reviews (9/12 apps), with 5 apps having more than 1000 reviews. Our exclusion criteria removed apps that required either a one-time payment or a subscription; however, we included 3 apps with subscription models since they offered free trials.

### Functionality Review Results

#### Overview

We now report on the findings from the functionality review, namely, the functionalities of tracking, visualizing, assessing, providing interventions, and ensuring privacy. Table 1 provides the main functionalities found in each app.

**Table 1.** Main functionalities that arose from the evaluation presented for each app.

Apps	Tracking	Visualizing	Providing interventions	Ensuring privacy
Bearable - Symptom Tracker	For tracking fatigue, symptoms, and associated factors	For visualizing tracked data	—^a^	For informing the user of their data handling
CareCircle - Health Platform	For tracking fatigue, symptoms, and associated factors	—	For social support	For informing the user of their data handling
Chronic Insights	For tracking fatigue, symptoms, and associated factors	For visualizing tracked data	—	For informing the user of their data handling
Curable: Chronic Pain Relief	—	—	For neuroeducation	For informing the user of their data handling
Elite HRV	For tracking associated factors	For visualizing tracked data	For energy estimation, psychoeducation	For informing the user of their data handling
Fibromyalgia Magazine	—	—	For psychoeducation	For informing the user of their data handling
Freeme: ME/CFS and Long COVID	—	—	For neuroeducation	For informing the user of their data handling
Guava: Health Tracker	For tracking fatigue, symptoms, and associated factors	For visualizing tracked data	—	For informing the user of their data handling
Gupta Program Brain Retraining	—	—	For neuroeducation, social support	For informing the user of their data handling
Turnto - Daily Breakthroughs	—	—	For social support, psychoeducation	For informing the user of their data handling
Visible: Pacing for illness	For tracking fatigue, symptoms, and associated factors	For visualizing tracked data	For energy estimation, psychoeducation	For informing the user of their data handling
Wave Health: Treatment Journal	For tracking fatigue, symptoms, and associated factors	For visualizing tracked data	—	For informing the user of their data handling
icompanion: understand your MS	For tracking fatigue, symptoms, and associated factors	For visualizing tracked data	For psychoeducation	For informing the user of their data handling
Ada - check your health	For tracking fatigue, symptoms, and associated factors	—	—	For informing the user of their data handling
SpoonieDay	For tracking associated factors	For visualizing tracked data	For pacing and energy estimation	For informing the user of their data handling
ME/CFS Pacing	For tracking associated factors	For visualizing tracked data	For pacing	—
Juva Stress and Migraine	For tracking associated factors	For visualizing tracked data	For biofeedback	For informing the user of their data handling

a Functionalities that are not available in the apps.

#### Tracking Fatigue, Symptoms, and Associated Factors

##### Overview

In this section, we explore the subfunctionalities that emerge under the main functionality of tracked content. At the first level of branching, we see fatigue tracking, symptom tracking, the tracking of associated factors and interventions, and finally modalities of the listed data as seen in Multimedia Appendix 4. The codes for the functionalities are also provided in Multimedia Appendix 5.

##### Tracking Fatigue

An important outcome is that from the 17 top-rated fatigue apps that we reviewed, less than half track fatigue (8 apps), and most of these (6 apps) track general fatigue, with the remaining 2 tracking physical fatigue. In addition, none of the apps offer a means of tracking fatigue automatically. This is important, indicating the potential of extending support for self-reporting of fatigue, and extending the measures of general fatigue to reflect other dimensions of fatigue: physical, cognitive, and mental or emotional. The apps that support self-reporting of fatigue do so through measures of trait fatigue or fatigue extended over time (5 apps), and state fatigue as fatigue momentarily experienced at the time of measurement (4 apps). The lack of usage regarding measures for state fatigue from half of the apps is also surprising, given the affordance of mobile apps for prompting its capture and thus tracking it through time.

**Figure 2.**
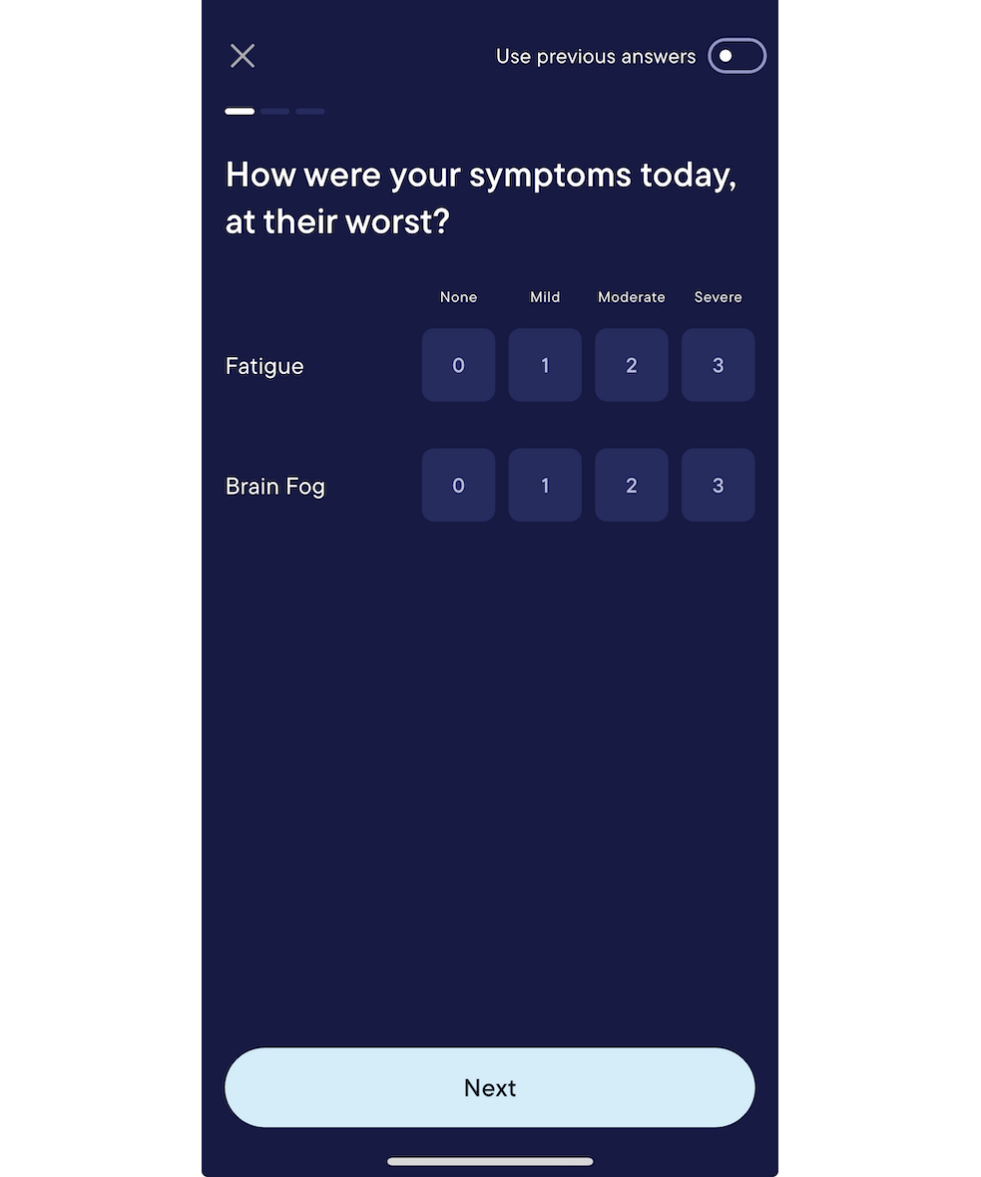
A screenshot showing a Likert scale used to capture data to track fatigue and other symptoms such as brain fog (image used with permission from the app’s developer).

Tracking fatigue is of great value to users with chronic fatigue, as this can be used to identify energy limits for the intervention of pacing [9] and thus, we believe the method of data capture and its granularity are important to be represented. To this end, we highlight that the few scales of fatigue used by the apps involve 1 item for each one, captured through 4-point (1 app) as seen in Figure 2 from the Visible: Pacing for illness app (Visible Health Inc) [46], 5-point (2 apps), 7-point (1 app), 10-point (1 app), and 11-point (1 app) Likert scales, numerical scales (1 app), that is, 0.0‐10.0 and semantic differential scales (1 app), that is, 3-point: mild, moderate, and severe level. From the apps that track fatigue only as a state, the scales can be completed at least once per day with no maximum (3 apps). No apps provide information on the validity of the used fatigue scales. Future app designs could better support ecological momentary assessment of fatigue states by using validated measures of state fatigue involving both general fatigue and its physical, cognitive, and mental aspects [2,47,48].

Besides fatigue, apps also track fatigue-related constructs such as energy level (4 apps), although there is surprisingly limited support for tracking exertion (2 apps) or crashes (1 app), which are essential for anticipating PEM and are much needed for managing chronic fatigue [49]. Each of these constructs is captured manually, for energy levels through 5-point Likert scales (3 apps) and a semantic differential scale with 5 levels (1 app), exertion through 4-point Likert scales (2 apps), and crashes (an incidence of PEM) through a yes or no check augmented with a text entry for additional notes. Regarding the frequency option for daily manual entering of energy levels, this can be exactly once (1 app), at least once per day (2 apps), or 3 times per day (1 app). Interestingly, these self-reported measures of energy, exertion, and crashes can benefit from being contextualized with spatiotemporal and activity data [2], which may be automatically tracked. However, few apps (3 apps) supported such integration of self-reported and automatically tracked measures related to fatigue, and from the data mentioned, only activity data were leveraged.

##### Fatigue Symptoms

Findings indicate rich support for tracking 4 main types of fatigue symptoms: cognitive (8 apps)**,** emotional (8 apps), physical (8 apps), and behavioral symptoms (5 apps). Multimedia Appendix 1 provides the top 40 most commonly tracked symptoms.

Most commonly tracked cognitive symptoms include brain fog (5 apps), difficulty with concentration (6 apps), or remembering (3 apps). Apps tracking emotional symptoms target mostly anxiety (8 apps), depression (7 apps), irritability (4 apps), pessimism (3 apps), sense of dread (3 apps), discouraged mood (1 app), disconnection or detachment from oneself and the surrounding world (ie, derealization or dissociation; 3 apps).

An important outcome is the prevalence of and range of bodily symptoms tracked by most apps. Such symptoms include most commonly pain (8 apps) and sensory symptoms (8 apps) such as numbness (6 apps) and sensitivity to light (6 apps), followed by specific ones concerning skin (6 apps), circulatory (7 apps), digestive (7 apps), muscular (5 apps), ocular (6 apps), respiratory (7 apps), reproductive (6 apps), or urinary systems (5 apps). Thus, most apps support a rich set of symptoms that could be tracked, which ranges between 76 and 155 different symptoms. While useful, tracking many symptoms, particularly manually, can be taxing [2], especially since many apps provide limited support for reducing such burden.

An interesting outcome is the various behavioral symptoms, with the most commonly tracked ones including nail biting (4 apps), restlessness (4 apps), fidgeting (3 apps), impulsivity (3 apps), skin picking (2 apps), and stimming (2 apps). A closer look at these indicates they are predominantly repetitive body movements [50], which previous work described as supporting self-regulation of sensory sensitivity in autism spectrum disorders [51], albeit also contributing to autistic fatigue [52].

##### Symptom Checkers

While the large number and diversity of the symptoms available to track is useful, it can also be overwhelming. Two apps use symptom checkers such as Ada’s symptom assessment functionality and Isabel (used in the CareCircle – Health Platform app) for supporting users to report their symptoms. While developed to support clinician diagnosis, symptom checkers implemented on mobile apps have also started to be explored by researchers investigating user perspectives for usability and acceptability [53,54]. Recent work has also explored AI-augmented symptom checkers with findings showing effective diagnosis, but problematic triage, and thus their use by unsupervised patients may be harmful [53].

##### Factors Associated With Fatigue

The reviewed apps also support tracking of additional factors which may impact fatigue and thus may allow people to better understand their fatigue experiences. Such factors include health data (7 apps) such as manually entered medical information (4 apps), medication intake (6 apps), or acute conditions such as flu and cold (1 app). From these, medication is tracked either by automatic logging after a one-off entry (3 apps) or through manual logging with either daily entries (1 app) or recording in a list (2 apps). Other health data were also tracked through the same list style of recording (4 apps). The modality of data capture is interesting from an HCI perspective, as HCI research on ELCs has called for tracking apps with a low impact on users’ energy levels [2,9]. In addition, since these users most likely have other symptoms alongside fatigue [55], this becomes particularly important. This opens up design opportunities toward implementing automatic tracking methods, as it would be the least taxing for this demographic. Health data were also automatically tracked, such as heart rate (6 apps), HRV (5 apps), resting heart rate (3 apps), oxygen saturation (2 apps), or breathing rate (2 apps). For the capture of health data, some apps leveraged the OS’s health app (6 apps), while other apps made use of the phone camera (1 app) or additional wearables such as heart monitors or smartwatch sensors.

Apps tracking physical activity focused mostly on step count (6 apps) or exercise duration (4 apps), captured automatically (6 apps), directly from the OS’s health app (6 apps), while 4 apps provide the option to manually enter step count. Mood was another important associated factor (6 apps), captured through a 5-point Likert scale (3 apps), augmented with emotion tags (1 app), a 10-point Likert scale with emotion tags (1 app), or merely a dropdown emotion list (1 app).

Another associated factor was sleep, captured through manually entered data using a 4- or 5-point Likert scale for sleep quality (1 app each), a dropdown list of factors impacting sleep (1 app), a time picker for daily entry of sleep duration (2 apps), or automatically tracked through the OS’s health app (2 apps). Other associated factors include weight (4 apps) and lifestyle data such as stress, caffeine consumption, or alcohol consumption (3 apps).

Moreover, a total of 5 apps offer the option to track well-being activities such as journaling (3 apps), meditation (3 apps), mindfulness (3 apps), and yoga (3 apps).

The use of scales may result in the loss of contextual data [56], which may aid sense-making; however, some apps have attempted to remedy this by offering the option for notes (5 apps) with each recording of most or all data types. The optionality of this feature is important, as this lowers the burden on users by allowing them to keep their data collection simple if their energy reserves are low.

### Visualizing Tracked Data

#### Overview

The daily use of the apps during the expert evaluation provided a sufficient amount of data for the assessment of their visualization functionalities. The findings from this reveal the provision of visualizations of tracked fatigue, symptoms, and associated data through charts representing different types of data, color-coded symptom severity, temporal aspects, and data summaries (Multimedia Appendices 6 and 7).

#### Types of Charts for Visualizing Fatigue

Findings show that the most common charts for fatigue visualization are line charts (4 apps) and bar charts (2 apps), although not all apps visualized tracked fatigue data (2 apps), despite users’ interest in understanding their tracked fatigue for the purpose of self-management [9].

#### Types of Data Represented in Charts

Fatigue is known to have numerous factors [2,9], and for this reason, users will want to reflect on other data sources as well. Findings indicate that 9 apps provide visualizations of a single data type, such as fatigue scores (6 apps). In addition, as the factors are known to be interdependent [2,9], users would benefit from being able to compare the data on the same chart. To this end, apps also offer visualizations integrating different tracked data (7 apps): most often fatigue data + symptom data (4 apps), fatigue data + health data (4 apps), health data + health data (5 apps), symptom data + symptom data (4 apps), symptom data + health data (4 apps), fatigue data + mood data (3 apps), symptom data + mood data (3 apps), physical condition + mental condition (1 app), or morning + evening mood (1 app). It was also noted that 3 of these apps providing visualizations of combined data do not have the option of integrating fatigue, which may limit users’ ability to better understand their fatigue by discerning factors that could affect it [9].

**Figure 3.**
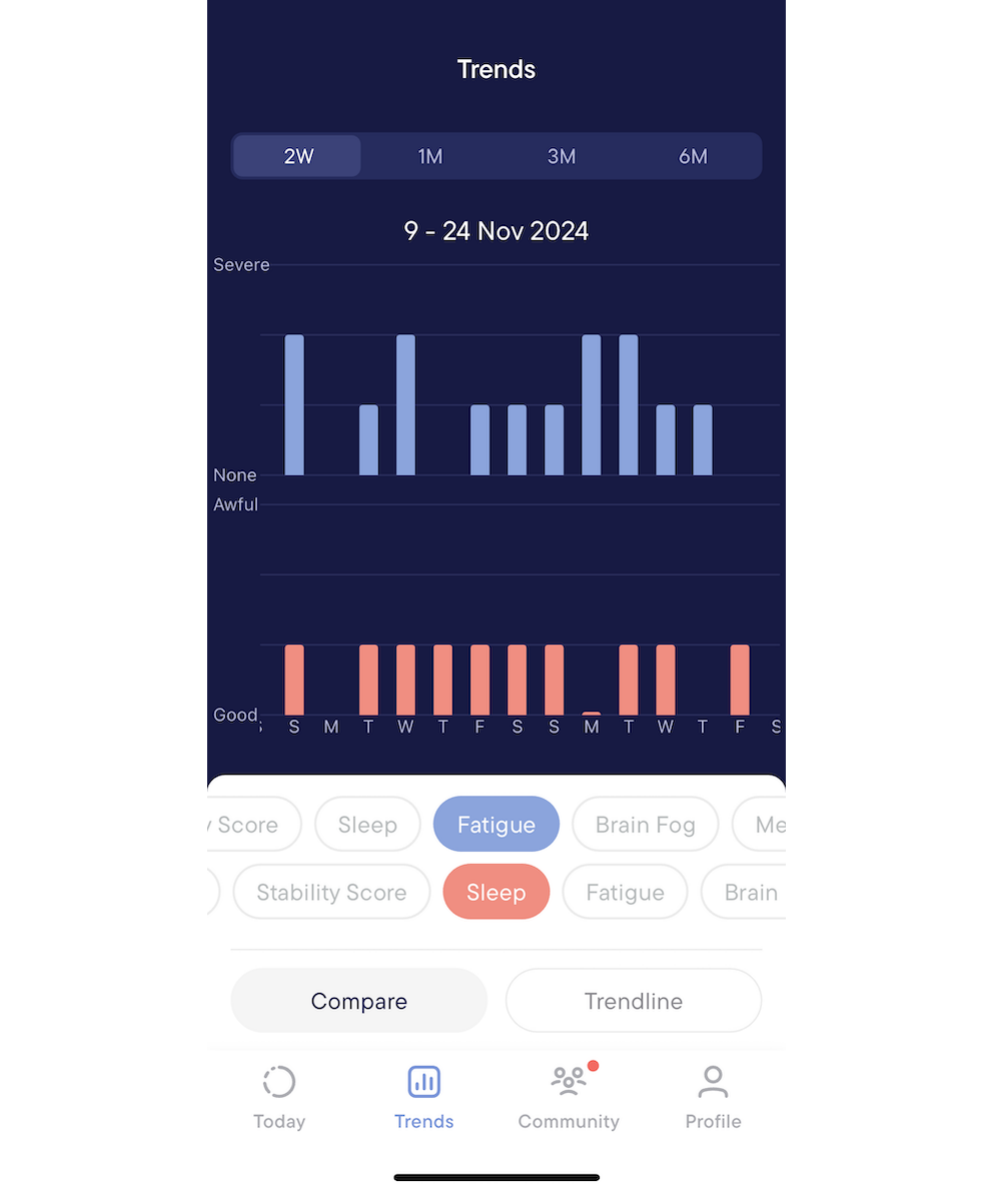
Screenshot showing a combined visualization of 2 *y*-axis variables for comparison (image used with permission from the app’s developer).

With respect to the number of different data used for such combined visualizations, most apps involve only 2 *y*-axis variables per visualization (6 apps), while 1 app allows an unlimited number of variables integrated in one app, which for numbers larger than 3 may be difficult to understand if not designed in an effective manner. Combined visualizations are often represented through multiple line charts, that is, one line for each *y*-axis variable (4 apps), combined bar and line charts (3 apps), combined scatter charts (1 app), parallel bar charts (1 app) as seen in Figure 3 from the Visible: Pacing for illness app [46], combined bar charts (1 app), and bar and dot combo charts (1 app). Of these, the most frequently used visualization was found to be combined line charts (4 apps). Given the interdependency of various aspects of fatigue [2,9], it is surprising that these visualizations do not look at them altogether in contrast to the isolated view, and this could be an area which future work could investigate. Finally, findings highlight that 1 app does not show combined visualizations, despite tracking different data.

#### Use of Color-Coded Symptom Severity in Visualizations

From the 8 apps that provide visualizations for symptom tracking, half of the apps use warm colors such as red to indicate negative symptoms or high severity level (4 apps), while the others do not use such color codes (4 apps). The former highlights severity level, which can increase one’s concerns and be perceived as judgmental [9].

#### Temporal Aspects of Visualizations: Timescale and Historic Versus Real-Time Data

All 9 apps providing visualizations for historic data do so with a range of timescale options such as the last 7 days (1 app), last 2 weeks (1 app), last month (3 apps), last 2 months (2 apps), last 3 months (3 apps), or a custom timescale ranging from the last 10 weeks to the last 7 years (1 app), any 7 days (3 apps), any 2 weeks (1 app), any month (2 apps), any year (1 app), as well as a custom scale with a start and end at any 2 points in time (1 app). These various timescales are beneficial, with the most recent past supporting understanding while leveraging the memory of recent events impacting the displayed fatigue scores, and the far past supporting understanding broader patterns of how fatigue changes over time [57]. Moreover, findings show that 7 apps display only historic data, 1 app displays both historic and real-time data, and another app displays only real-time data. From the 2 apps that show real-time data, both apps use it for biofeedback interventions. In addition, the real-time data are measured with an HRV monitor for one app and with both a camera and an accelerometer for the other. Finally, one app visualizes HRV, and the other visualizes heart rate and breathing rate.

From the apps that visualized fatigue, we note that 2 apps represented all data points per day on the chart, while the other 4 apps represented one data point per day. While 2 of the latter only record 1 fatigue score per day, the other apps either average the score (Guava: Health Tracker) or show the highest score for the day (Bearable - Symptom Tracker). This may be either a benefit or a drawback to self-trackers who deal with chronic fatigue. On one hand, representing all the data points could take more mental effort to digest, which could be remedied by per-day summaries; however, they hide the changes in fatigue, diminishing the insights obtainable from their tracking efforts. This highlights the value of visualizing a minimum of 3 data points a day, with options to show fatigue data before and after activities, as users typically seek to understand the impacts of activities for pacing purposes [9].

#### Presentation of Distinct Summaries of Some or All Data

Findings reveal that 4 apps display data summaries. One app shows averages of any tracked variable within a chosen timescale. A second app shows the averages of symptoms and steps over a weekly or monthly period. A third app shows averages of morning and evening mood over 7, 30, or 90 days. The fourth app shows averages of daily scores within a yearly view visualization, and averages of monthly scores within a monthly view. These data summaries are important, as providing users a more condensed form of data could reduce the cognitive load of understanding their fatigue, which is important in preventing the apps from exacerbating their condition [2].

### Assessment Tools

Regarding symptoms, 2 apps provide valid scales to assess the symptoms’ impact on everyday life (Multimedia Appendices 8 and 9). One is the Functional Capacity in ME/CFS (FUNCAP) [58] (David Davies-Payne), whose development was informed by patients living with fatigue. This tool effectively accounts for the impact of PEM on completing activities. In its short version, it consists of 27 items covering 8 domains from personal hygiene to concentration. The other tool is Quality of Life in Neurological Disorders (Neuro QOL) [59,60], consisting of 8 or 9 items for each of 13 domains from physical to mental health. Both tools are retrospective reports as they require reflection on past experiences to complete [61].

### Interventions for Managing Fatigue

#### Overview

**Figure 4.**
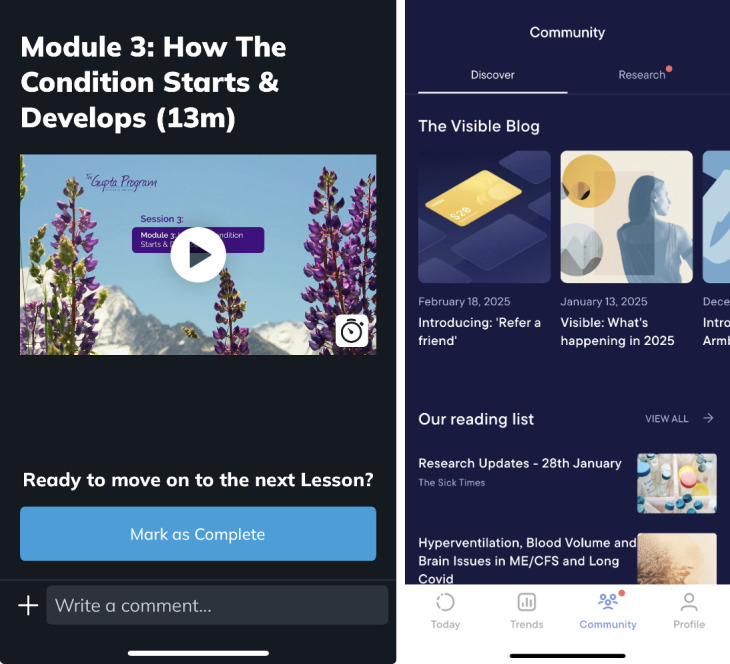
Screenshots showcasing interventions provided by 2 different apps (psychoeducation on the left, and educational support on the right [images used with permission from the apps’ developers]).

Despite the rich set of symptoms and tracked data, the top-rated apps for fatigue provide limited interventions for the self-management of fatigue, and in particular, those leveraging tracked data (Multimedia Appendices 10 and 11). Findings highlight a range of interventions provided by the apps, one of which is neuroeducation (3 apps); a treatment approach that aims to improve user well-being via educational lessons and exercises built around neuroscience concepts [62]. The apps in our review chose an approach centered on the concept of neuroplasticity and one such app is the Gupta Program Brain Retraining (Harley Street Solutions LTD) [63], as seen in Figure 4. Another intervention is psychoeducation (5 apps), which is delivered through articles informing users of their condition along with possible treatments and informing them of new advancements in research surrounding their condition, as seen in the Visible: Pacing for illness app [46] in Figure 4. Functionalities for social support (3 apps) were also implemented, in the form of community engagement through forum posts and social networking. These functionalities offer value to users with ELCs by providing them with a space for support while promoting discussion around their condition [7,10,15-17]. Moreover, 2 apps included biofeedback-based therapy, which aims to regulate the nervous system to reduce symptoms through the use of deep breathing (2 apps) or muscle relaxation (1 app) exercises, the latter of which has been known to reduce fatigue and may help individuals with CFS [64]. Finally, 2 apps were found to offer functionalities supporting pacing (2 apps).

Pacing is an important intervention, tailored for fatigue [1], which involves planning and allocating one’s energy toward activities to be completed based on their energy demands [9] through the so-called spoon management [9,65] or energy envelope approaches [9,66]. Pacing involves identifying what activities one can do, in addition to the extent that such activities can be carried out [67]; additionally, it entails stopping activities when breaching PEM and can also integrate the act of estimating energy levels [68]. Previous findings indicated the value of pacing interventions for managing fatigue in addition to preventing overexertion and PEM [1]. While not an intervention per se, 3 apps also provide support for energy estimates, which is important as it can provide input for pacing interventions.

#### Energy Estimation Using HRV and Pacing

**Figure 5.**
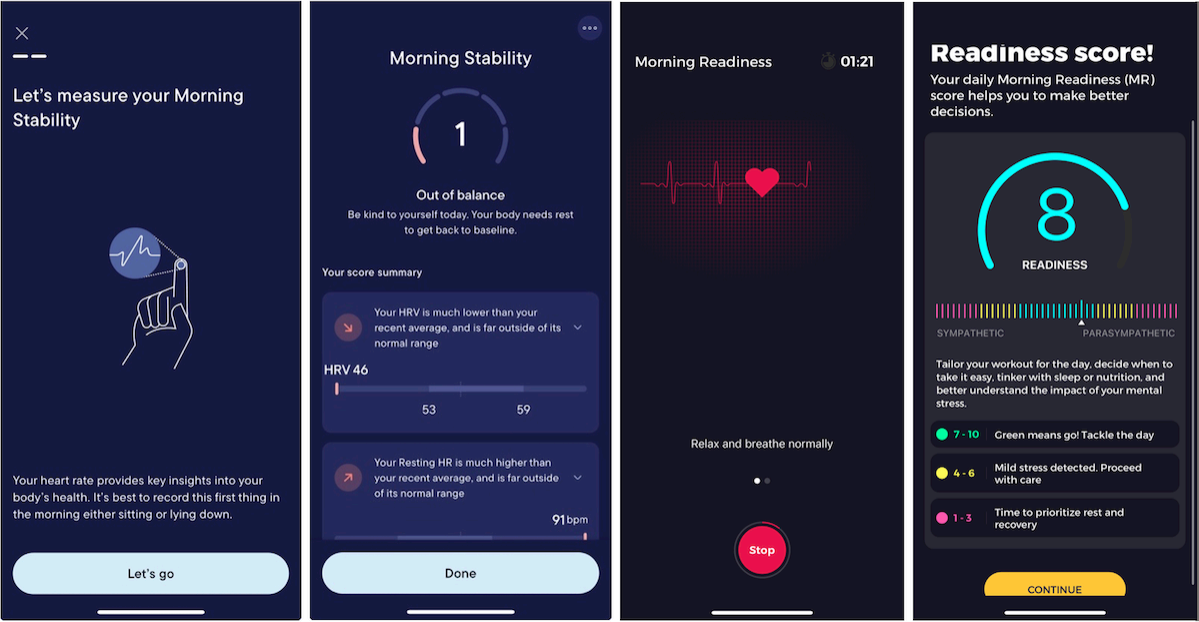
Screenshots showing energy estimation approaches by 2 different apps (images used with permission from the apps’ developers).

Estimates of energy are manually tracked through self-reports (1 app), which is the traditional approach as a prerequisite for pacing. Interestingly, another 2 apps use a method of tracking daily energy estimates through HRV measurement in the morning, which is compared to the user’s baseline to provide either a stability score by one app (Figure 5) [46], or a readiness score by the other (Figure 5) [69]. These scores are used to notify the user of potential flare-ups of their conditions and, as mentioned before, can inform their pacing decisions [68].

Previous work suggested the potential of HRV data as an indicator of fatigue [70,71], albeit its use to estimate energy has been less explored. Such approaches to estimate both fatigue and energy levels are crucial to lower the burden of tracking and its cognitive load, which is especially beneficial for an individual who may be approaching PEM [2]. Interestingly, however, not all apps estimating energy also provide pacing interventions.

#### Pacing Interventions

**Figure 6.**
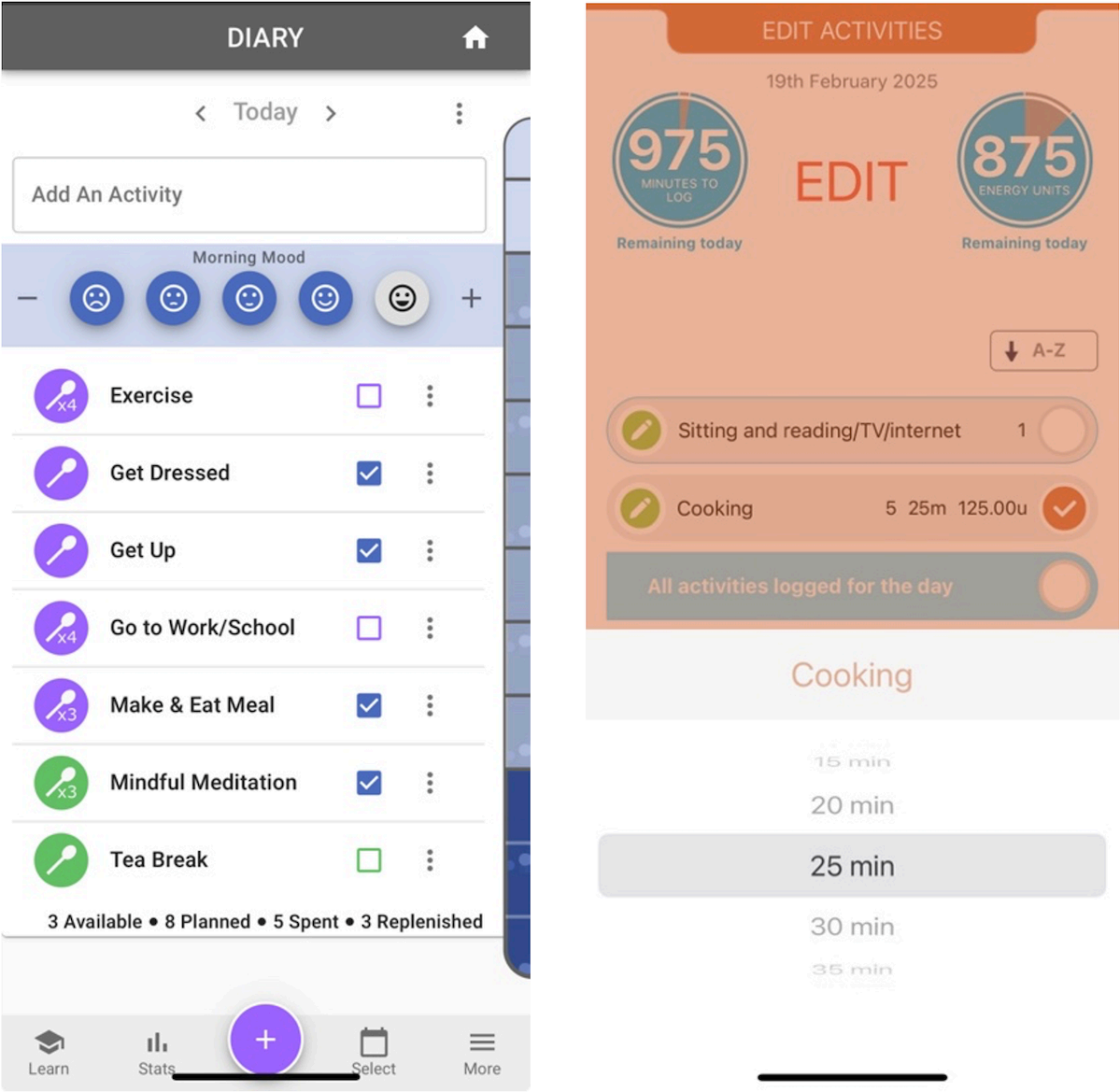
Screenshots from apps providing spoon management (left), and an energy envelope approach (right; images used with permission from the apps’ developers).

These interventions are provided only by 2 apps using different energy units. One app uses spoon management [9,65] as seen in Figure 6 [72], and the other leverages energy envelope approaches [9,66] as seen in Figure 6 [73]. The former has a maximum daily energy level set to 12 spoons, while the latter uses a less conventional and finer-grain maximum daily amount of 1000 energy units. Both apps provide predefined and customizable activity lists with predefined energy impacts for each of the listed activities, as well as gamification in the form of a limited-quantity source [74] to track and account for energy as a resource. Additionally, they both use traffic light–style red and green coding to visualize energy usage as positive and negative [75], and to deliver cautionary feedback as a consequence of extending one’s energy beyond the daily threshold amount.

Planning daily activities based on one’s energy can support not just pacing, but also structure and order, or routinization, with previous findings indicating its benefits for people living with CFS [9]. Routinization could be further supported by automatically prefilling in activity fields on apps to further reduce the user’s burden. This is important as a previous study has shown that fatigue symptoms can impede user engagement with self-management apps [9].

#### Theoretical and Empirical Underpinnings of Interventions

Our findings show that 6 of the apps offering interventions such as psychoeducation, neuroeducation, energy estimation, and biofeedback had provided either theoretical or empirical groundings of their methods and informed the user either through app help pages or the app’s website. From these, 4 of the apps cite academic work to support the efficacy of the intervention that they provide neuroeducation (Gupta Program Brain Retraining [Harley Street Solutions LTD]); pacing (Visible: Pacing for illness [Visible Health Inc.]), an energy estimation app; biofeedback (Juva Stress and Migraine [Juva Health Inc.]); and HRV-based interventions (Elite HRV [Elite HRV Inc]), respectively. However, only 2 of these apps (Visible: Pacing for illness [Visible Health Inc.] and Gupta Program Brain Retraining [Harley Street Solutions LTD]) provide empirical evidence supporting the effectiveness of the app itself. Two other neuroeducation apps (Freeme: ME/CFS and Long COVID [Freeme Health Ltd], and Curable: Chronic Pain Relief [Curable Inc]) cite academic work supporting all or some of the information provided to users in their educational material.

### Data Extracted From App Privacy Policies and Cookie Notices

#### Overview

We now report on data from app privacy policies and cookie notices through 3 main identified themes, namely the target age groups of the apps, information surrounding the handling of data, and the usage and purpose of cookies (Multimedia Appendices 12 and 13).

#### Target Age Group for App Use

Findings reveal that the majority of the apps (10 apps) restricted their use to nonadult individuals. From these, 7 apps mention that they do not provide services to users younger than 18 years, 2 apps do the same for users younger than 16 years, and 1 app restricted nonadult users unless the legal guardian or carer provides consent.

#### Information Surrounding the Handling of Data

Findings indicate that for 11 apps, their developers disclosed that they stored data gathered through the app on company servers, while 1 app stored it with a third-party site called Sentry Inc. The latter has stated in its privacy policy that this is done only for anonymized crash data.

As the apps store their data outside the user’s device, it is important to reveal the parties with whom the data are being shared. Findings show that via the use of the app, users consent to sharing data with advertisers for 4 apps, 2 of which have revealed that personal data will be shared. Other parties with whom data will be shared in this manner include analytics (3 apps), anonymous crash data processing as mentioned earlier (1 app), and research (2 apps), but in an anonymized form.

Outside of the previously mentioned data, some apps also offered the option to opt-in to third-party data sharing with advertisers (1 app), analytics (1 app), the OS’s health app (1 app), third-party backup storage providers (1 app), and 5 apps present the option to share data with researchers, of which 2 apps claimed that the data will be anonymized, and a third app revealed that the data will be used for product development and health research. It is worth noting that opting in with advertisers, the OS’s health app, and backup storage providers involves the sharing of personal data.

Another important finding is the information surrounding the sale of user data; 3 of the apps were found to sell data, one of which revealed that they only sell personal information with the user’s permission. Seven other apps offered no information surrounding the sale of data, and the remaining 6 apps revealed that they do not sell. Regarding the latter, 4 of them explicitly stated no, 1 app claimed that they do not sell the information covered by the policy, and the final app revealed that it does not sell data from Californian residents. Meanwhile, 2 of the 3 apps selling data informed that the data were being sold to advertisers (2 apps) and analytics providers (1 app). Data surrounding the sale of information were mostly easy to find, with the majority (6 apps) highlighting the information under clearly labeled headings and subheadings, while 2 apps detailed that they do not sell it without highlighting or obfuscating the information. However, one app that sold personal data did not state it on the main page of the privacy policy and was instead presented on a separate page for Californian and US residents. While this page was linked in the privacy policy, the document did not point to the information regarding the sales being there and thus appears hidden to users outside the United States, as they may not explore sections irrelevant to them. This emphasizes the importance of clearly highlighting information regarding sales to users through the use of subheadings to improve discoverability.

Regarding ownership of data, only 1 app’s privacy policy offered information surrounding this, and it revealed that the developer is the data owner.

#### Usage and Purpose of Cookies

We also analyzed the app’s privacy policies and cookie notices for information surrounding cookies and have found that 8 apps used cookies for analytics purposes, with one of the apps only enabling cookies outside of app and website areas requiring a log-in to use, to protect their users’ health data. A total of 6 other apps stated that they made use of cookies for both advertisement and analytics, while the remaining 3 provided no information surrounding the use of cookies.

## Discussion

### Principal Results

Within the main tracking functionality, our findings highlight a rich set of tracked content on fatigue, its symptoms, and associated factors, whose understanding is supported by various visualizations. Regarding interventions, however, a limited number of apps leveraged this rich and diverse tracked data to support pacing of estimated energy. In light of these outcomes, we articulate 5 design implications capturing both the strengths of some of the top-rated apps for fatigue, as well as how such apps can be further improved, along with the broader design of technologies for fatigue.

### Supporting Hybrid Tracking: Balancing Validity of Self-Reported Fatigue With Low Burden Automatic Tracking of Symptoms

Two key outcomes are the limited use of validated scales for capturing and tracking fatigue, which limits the validity of tracked data and its value for recommending interventions. Validated scales come with potential drawbacks regarding lower engagement due to their burden [56]. Information might also be less personally meaningful to the participant as the level of data collected is restricted to that elicited by the scale; however, the burden can be reduced by using scales that are short and simple [56]. In addition, the loss of meaningful contextual data can be remedied by offering the option to add notes with every entry, as 5 apps in this review have done. Therefore, we strongly recommend the use of validated fatigue scales, both for trait and state fatigue. The former can be used before and after an intervention program, while the latter can be used for frequent tracking of fatigue. To identify such valid scales, we can draw from a previous systematic review [76], showing that the most commonly used trait scale includes the Modified Fatigue Impact Scale (MFIS) [77], and for state scale we could use the 1-item Samn-Perelli Fatigue Checklist [78], or the 4-item Fatigue State Questionnaire (FSQ) [79], which is also used in ecological momentary assessment with digital technologies [80]. The latter state scales are not only valid but also consist of a small number of items, representing an important consideration for lowering user burden for daily tracking. Moreover, we have seen emerging efforts to capture fatigue through HRV data both in some of the apps via commercial heart rate sensors (which include chest straps such as the Polar H10) capable of HRV capture by the Elite HRV app, and photoplethysmography [81] conducted through the smartphone camera by the Visible: Pacing for illness app (Visible Health Inc.), along with scholarly work [70,71] typically focusing on electrocardiogram (ECG) data. If these approaches can be scaled to an automatic process, this could offer substantial support to users seeking to lower the burden of tracking. In addition, the automatic tracking of fatigue has even been found to be desired by participants in co-design workshops tackling pacing technologies [11]. The implementation of a hybrid tracking system using low-effort or even automatic capture of fatigue through both self-reported measures of scales and HRV data can explore and ensure the validity of the latter, while lowering the burden of the former [2]. For this, we can also think of novel, low-cost tracking methods on smartwatch interfaces, possibly ML-powered [2].

Findings also indicate the vast number of symptoms available to be tracked, which can be much needed to inform the management of fatigue, albeit they may be taxing to track and understand. To mitigate this downside, we propose the use of free-form conversational interfaces supported by natural language processing (NLP); this can reduce the mental load on users while also allowing for richer data input and capture [82]. We further suggest hybrid tracking of such symptoms, leveraging low-burden self-reported data with lower frequency, augmented by automatically tracked data [2,9] through the app, or phone itself, as well as smartwatch sensors via the OS’s health apps paired with heart rate monitors. We have also seen emerging efforts to integrate symptom checkers such as Ada and Isabel to support users in navigating their symptoms. This opens up new design opportunities for AI-augmented symptom checkers, although ethical implications need to be carefully considered, as despite their usability and acceptability [83], the value of AI checkers for unsupervised patients may be harmful [53].

### Contextualizing Fatigue Data With Symptoms and Associated Factors: Combined Visualizations for Sense-Making

Findings reveal that while most apps support the tracking and visualizing of fatigue data, only half support combined visualizations, some of which show fatigue data shown alongside other tracked symptoms or associated factors, while others combine different symptoms with or without associated factors, albeit without fatigue data. Combined visualizations highlighting fatigue data against symptoms or associated factors are, however, crucial for understanding if and how the latter may have impacted changes in state fatigue over time. Integrating different data types in one ready-to-understand visualization is not trivial. For this, we suggest low complexity ensured by combining fewer data types, such as fatigue and steps, for instance, through visualizations with 1 or 2 line charts augmented with additional information such as one’s location. The selection of data types, possibly powered by AI, would be best informed by data types with the strongest impact on the changes in fatigue scores. Visualizations may also include data summaries over time, some already supported by a small number of apps. Color-coded symptom severity levels may be avoided to lower users’ emotional concerns, and more subtle ways of informing severity levels can be instead explored. With respect to timeline, we suggest providing options with complementary value: visualizations of the most recent past benefit from memory of recent events to support recall of key factors impacting fatigue and changes in daily scores, while those of the far past can highlight broader patterns of changes in fatigue over time [57].

### Normalizing the Inclusion of Energy Estimates and Pacing Interventions in Apps Targeting Fatigue

While many of our reviewed apps support interventions, only a few provide support for the intervention of pacing. This is surprising, given the rich body of work indicating its effectiveness for the self-management of fatigue [2]. This opens up a design space for pacing interventions for which we suggest drawing from spoon management [9,65] or energy envelope approaches [9,66]. We can think of novel interfaces which leverage the spoons concept as energy units for energy estimates, scaffold users’ planning of energy estimates to daily activities, and provide subtle rather than punitive guidance when an energy threshold is exceeded to help users prevent crashes. The latter is important, as crashes can cause people’s energy levels to remain depleted over time, that is, days [2] or even months [49]. As shown by the apps “SpoonieDay” (Blackburn Labs) and “ME/CFS Pacing” (Emerge Australia Inc), one approach to mitigate this is for users to identify at the start of the day their available overall energy, prioritize the main activities that can be covered with it, and allocate the available energy toward core activities, while monitoring that their expenditure remains within the allocated limits. Although users can try to estimate this via methods such as spoon management [9,65] and energy envelope approaches [9,66], they may still underestimate their energy usage needs, and therefore, a warning system may be more useful.

Planning daily activities based on one’s energy can support not just pacing, but also structure and order, or routinization, with previous findings indicating its benefits for people living with CFS [9]. Routinization could be further supported by automatically prefilling in activity fields on apps to further reduce the user’s burden. This is important as a previous study has shown that fatigue symptoms can impede user engagement with self-management apps [9].

We have also noted the limited use of tracking crashes (PEM events), which can be highly useful, as these happen when participants go over their limits. Identifying one’s own limits is one of the foundational aspects of pacing, and this can be further supplemented by contextualizing the data with activity data and spatiotemporal information.

Most importantly, findings revealed that the apps that offer pacing functionalities do not provide energy estimation functionalities, and vice versa. Users would benefit from these functionalities being put together, as energy estimation would give them more information to work with for pacing, and could especially support decision-making (when planning activities) on days when they are more cognitively fatigued.

### Comparisons With Prior Personal Informatics Research in the Space of Chronic Conditions

We compared the findings to those of previous works in the field of personal informatics, as they can serve as valuable evidence given the common ground they share. The main difference that we noted is that the apps we reviewed track a much larger range of symptoms and health data compared to the works in the space of personal informatics. Due to fatigue being impacted by a wide range of interrelated factors [2,9], this level of tracking could be needed by users to understand their condition. While tracking a substantial number of symptoms will eventually overwhelm users [2], lightweight tracking solutions, as discussed earlier, could help negate this.

With regard to interventions, pacing is a unique approach that has received some focus from the field of personal informatics. For the purpose of pacing, while historic data are useful, Felipe et al [29] reported that users with chronic pain desired to see their data in real-time to identify if they were exceeding their limits. However, we have found that only a limited number of apps support this feature. Future apps could implement functionalities to show a live view of tracked data, as this could be useful for identifying if users are approaching PEM.

### Limitations

The main limitations of the study are that our app review did not include non-English apps, apps outside the UK app stores, and finally, paid apps. Regarding the latter, as mentioned earlier, and aligned with previous work [4,33,35,40], we focused on apps that were either completely free or offered free trials because, in the health care context, accessibility and fairness are vital, especially since vulnerable users may not be able to afford subscriptions [41]. Nevertheless, other apps such as paid ones, non-English apps, and apps outside the United Kingdom are indeed likely to include additional valuable features, and future work could explore these to identify any further design insights on supporting the management of fatigue via apps.

### Conclusions

This paper presents a functionality review of 17 fatigue apps selected as top-rated apps from the Google Play Store and the Apple App Store. Findings indicate 3 main functionalities pertaining to tracking fatigue, its symptoms, and associated factors, visualizing tracked content, and providing interventions for the self-management of fatigue. Our findings informed 3 implications for sensitive design of technologies for people living with fatigue, which include supporting hybrid tracking to balance the validity of self-reported fatigue with low-burden automatic tracking of symptoms, contextualizing fatigue data with symptoms and associated factors through combined visualizations for sense-making, and normalizing the inclusion of energy estimates and pacing interventions in apps targeting fatigue.

## Supplementary material

10.2196/84755 Multimedia Appendix 1 Codebook for the full set of codes, including main codes and all subcodes under the main functionalities of tracking, visualizing, assessing, providing interventions, and ensuring privacy.

10.2196/84755 Multimedia Appendix 2 Apps obtained from searching the Apple App Store and the Google App Store with the keywords “chronic fatigue syndrome”, “fatigue”, “ME/CFS”, “CFS,” “myalgic encephalomyelitis,” and “pace or pacing.”

10.2196/84755 Multimedia Appendix 3 Characteristics of apps selected for review.

10.2196/84755 Multimedia Appendix 4 Frequency of codes for tracking functionality.

10.2196/84755 Multimedia Appendix 5 Codes for tracking functionality.

10.2196/84755 Multimedia Appendix 6 Frequency of codes for visualization functionality.

10.2196/84755 Multimedia Appendix 7 Codes for visualization functionality.

10.2196/84755 Multimedia Appendix 8 Frequency of codes for screening and assessment functionality.

10.2196/84755 Multimedia Appendix 9 Codes for screening and assessment functionality.

10.2196/84755 Multimedia Appendix 10 Frequency of codes for intervention functionality.

10.2196/84755 Multimedia Appendix 11 Codes for intervention functionality.

10.2196/84755 Multimedia Appendix 12 Frequency of codes for privacy features.

10.2196/84755 Multimedia Appendix 13 Codes for privacy features.

## Abbreviations

CFS: chronic fatigue syndrome
ECG: electrocardiogram
ELC: energy-limiting condition
FSQ: Fatigue State Questionnaire
HCI: human-computer interaction
HRV: heart rate variability
IoT: Internet of Things
MFIS: Modified Fatigue Impact Scale
ML: machine learning
Neuro QOL: Quality of Life in Neurological Disorders
NLP: natural language processing
PEM: postexertional malaise
PRISMA: Preferred Reporting Items for Systematic Reviews and Meta-Analyses
VR: virtual reality
