# Supplementary material for: Functionality Review of Mobile Apps for the Tracking and Self-Management of Fatigue: Systematic Search in App Stores and Content Analysis
Source: JMIR Mhealth Uhealth. 2026 Aug 11;14:e84755. doi: 10.2196/84755 (PMC13460801; doi:10.2196/84755)
Supplement: Supplementary file 1 [file mhealth-v14-e84755-s004.docx]

Distribution of codes for tracking functionality, some of the items in the list are not mutually exclusive.

| Main codes | Sub-codes level 1 | Sub-codes level 2 | Sub-codes level 3 | App numbers (/17) |
| --- | --- | --- | --- | --- |
| What is tracked? | Fatigue  Fatigue symptoms  Associated factors    Custom variables | Fatigue category  Fatigue Trait/State  Energy level  Exertion  Crashes  Cognitive symptoms  Emotional symptoms  Behavioral symptoms  Physical symptoms  Physical activity  Mood  Sleep  Health  Lifestyle factors  Wellbeing options | General fatigue  Physical fatigue  N/A  Trait  State  Trait + State  Physical pain symptoms  Sensory symptoms  Skin symptoms  Circulatory symptoms  Digestive symptoms  Muscular symptoms  Ocular symptoms  Respiratory symptoms  Reproductive symptoms  Urinary symptoms  Other symptoms  Step count  Exercise duration  Health data  Medical information  Medication intake  Acute conditions | 6 (35%)  2 (12%)  9 (53%)  4 (24%)  3 (18%)  1 (6%)  4 (24%)  2 (12%)  1 (6%)  8 (47%)  8 (47%)  5 (29%)  8 (47%)  8 (47%)  6 (35%)  7 (41%)  7 (41%)  5 (29%)  6 (35%)  7 (41%)  6 (35%)  5 (29%)  8 (47%)  6 (35%)  4 (24%)  6 (35%)  5 (29%)  7 (41%)  4 (24%)  6 (35%)  1 (6%)  3 (18%)  5 (29%)  3 (18%) |
