# Supplementary material for: Functionality Review of Mobile Apps for the Tracking and Self-Management of Fatigue: Systematic Search in App Stores and Content Analysis
Source: JMIR Mhealth Uhealth. 2026 Aug 11;14:e84755. doi: 10.2196/84755 (PMC13460801; doi:10.2196/84755)
Supplement: Supplementary file 2 [file mhealth-v14-e84755-s006.docx]

Distribution of codes for visualization functionality, some of the items in the list are not mutually exclusive.

| Main codes | Sub-codes level 1 | Sub-codes level 2 | Sub-codes level 3 | App numbers (/17) |
| --- | --- | --- | --- | --- |
| Visualization | Type of chart for fatigue  Individual/combined tracked content  Color-coded symptom severity  Temporal aspects  Distinct summaries of some or all data | Data combinations  Combination count  Visualization chart used for combined  Red  No colour-coding  N/A  Timescale  Historic data  Real-time data  Any data/any timescale  Symptom, steps/week, month  Morning, evening mood/week, month  Daily, monthly scores/month, year | Line  Bar  None  Fatigue data + symptom data  Fatigue data + mood data  Fatigue data + health data  Symptom data + symptom data  Symptom data + mood data  Symptom data + health data  Health data + health data  Physical condition + mental condition  Morning + evening mood  No fatigue comparison  2  2-unlimited  Bar and line combo chart  Combined line chart  Combined scatter chart  Parallel bar chart  Combined bar chart  N/A | 8 (47%)  2 (12%)  2 (12%)  4 (24%)  3 (18%)  4 (24%)  4 (24%)  3 (18%)  4 (24%)  5 (29%)  1 (6%)  1 (6%)  3 (18%)  6 (35%)  1 (6%)  3 (18%)  4 (24%)  1 (6%)  1 (6%)  1 (6%)  10 (59%)  4 (24%)  4 (24%)  9 (53%)  9 (53%)  9 (53%)  2 (12%)  1 (6%)  1 (6%)  1 (6%)  1 (6%) |
