# Supplementary material for: Functionality Review of Mobile Apps for the Tracking and Self-Management of Fatigue: Systematic Search in App Stores and Content Analysis
Source: JMIR Mhealth Uhealth. 2026 Aug 11;14:e84755. doi: 10.2196/84755 (PMC13460801; doi:10.2196/84755)
Supplement: Supplementary file 3 [file mhealth-v14-e84755-s008.docx]

Distribution of codes for screening & assessment functionality, some of the items in the list are not mutually exclusive.

| Main codes | Sub-codes level 1 | Sub-codes level 2 | Sub-codes level 3 | App numbers (/17) |
| --- | --- | --- | --- | --- |
| Screening & Assessment | Screening tools  Assessment tools | Isabel Symptom Checker  Ada Symptom Assessment  FUNCAP27 Questionnaire  Neuro-Qol questionnaires |  | 1 (6%)  1 (6%)  1 (6%)  1 (6%) |
