# Supplementary material for: Functionality Review of Mobile Apps for the Tracking and Self-Management of Fatigue: Systematic Search in App Stores and Content Analysis
Source: JMIR Mhealth Uhealth. 2026 Aug 11;14:e84755. doi: 10.2196/84755 (PMC13460801; doi:10.2196/84755)
Supplement: Supplementary file 4 [file mhealth-v14-e84755-s010.docx]

Distribution of codes for intervention functionality, some of the items in the list are not mutually exclusive.

| Main codes | Sub-codes level 1 | Sub-codes level 2 | Sub-codes level 3 | App numbers (/17) |
| --- | --- | --- | --- | --- |
| Interventions | Pacing  Energy estimation  Factors affecting fatigue  Neuroeducation  Psychoeducation  Social support  Biofeedback  Theoretical/Empirical Underpinnings | Planning  Planning & tracking  Energy measurement  Maximum daily energy  Maximum weekly energy  Penalty of going over limits  Activity list  Impact from each activity  Method  Subjective reporting of energy levels  Objective reporting of energy levels  Measurement (for objective)  Daily frequency (for objective) | Spoon tracking  Energy units  12 spoons  1000 energy units  Spoons borrowed from next day  Less weekly energy for other days | 1 (6%)  1 (6%)  1 (6%)  1 (6%)  1 (6%)  1 (6%)  1 (6%)  1 (6%)  1 (6%)  2 (12%)  2 (12%)  3 (18%)  1 (6%)  2 (12%)  2 (12%)  2 (12%)  2 (12%)  3 (18%)  5 (29%)  3 (18%)  2 (12%)  6 (35%) |
