# Supplementary material for: Functionality Review of Mobile Apps for the Tracking and Self-Management of Fatigue: Systematic Search in App Stores and Content Analysis
Source: JMIR Mhealth Uhealth. 2026 Aug 11;14:e84755. doi: 10.2196/84755 (PMC13460801; doi:10.2196/84755)
Supplement: Supplementary file 5 [file mhealth-v14-e84755-s012.docx]

Distribution of codes for privacy features, some of the items in the list are not mutually exclusive.

| Main codes | Sub-codes level 1 | Sub-codes level 2 | Sub-codes level 3 | App numbers (/17) |
| --- | --- | --- | --- | --- |
| Privacy | Data storage  Data selling  Child protection  Data sharing  Cookies use | Company servers  Other  Yes  No  No mention  Does not provide service to users under 16  Does not provide service to users under 18  Legal guardian should provide consent  Consented through use of app  Opt-in  Analytics  Advertisement & analytics  No mention | Advertisement  Analytics  Crash data processing  Research (academic, clinical, educational, informational, medical, and other purposes such as product development  Research (unspecified)  Advertisement  Analytics  OS health app  Research (unspecified in privacy policy)  Research (product development and health research)  Back-up/storage providers (third party) | 11 (65%)  1 (6%)  3 (18%)  6 (35%)  7 (41%)  2 (12%)  7 (41%)  1 (6%)  4 (24%)  3 (18%)  1 (6%)  1 (6%)  1 (6%)  1 (6%)  1 (6%)  1 (6%)  4 (24%)  1 (6%)  1 (6%)  8 (47%)  6 (35%)  3 (18%) |
